# Comparison of cusp-overlap projection and standard three-cusp coplanar view during self-expanding transcatheter aortic valve replacement: A systematic review and meta-analysis

**DOI:** 10.3389/fcvm.2022.927642

**Published:** 2022-08-17

**Authors:** Yujing Chen, Gangjie Zhu, Xin Liu, Weilin Wu, Hui Chai, Minjie Tao, Dongmei Kong, Yingzi Li, Li Wang

**Affiliations:** ^1^Department of Hepatobiliary Surgery, Affiliated Hangzhou First People’s Hospital, Zhejiang University School of Medicine, Hangzhou, China; ^2^Department of Cardiology, Affiliated Hangzhou First People’s Hospital, Zhejiang University School of Medicine, Hangzhou, China; ^3^Department of Endocrinology, Affiliated Hangzhou First People’s Hospital, Zhejiang University School of Medicine, Hangzhou, China

**Keywords:** cusp-overlap projection, fluoroscopy, permanent pacemaker implantation (PPI), implantation depth, transcatheter aortic valve replacement, TAVR

## Abstract

**Objective:**

Permanent pacemaker implantation (PPI) is a common complication after transcatheter aortic valve replacement (TAVR). Recently, the cusp-overlap projection (COP) technique was thought to be a feasible method to reduce PPI risk. However, the evidence is still relatively scarce. Therefore, this meta-analysis was performed to compare COP and standard three-cusp coplanar (TCC) projection technique.

**Methods:**

PubMed and EMBASE databases were systematically searched for relevant literature published from the inception (EMBASE from 1974 and PubMed from 1966) to 16 April 2022, following the Preferred Reporting Items for Systematic Reviews and Meta-Analyses (PRISMA) statement. The primary outcome of interest was post-operative (including in-hospital and 30-day) PPI.

**Results:**

Total of 3,647 subjects from 11 studies were included in this meta-analysis. Of those, 1,453 underwent self-expanding TAVR using COP and 2,194 using TCC technique. In a pooled analysis, the cumulative PPI incidence was 9.3% [95% confidence interval (CI): 6.9–11.7%] and 18.9% (95% CI: 15.5–22.3%) in the COP group and TCC group, respectively. The application of the COP technique was associated with a significant PPI risk reduction (*I^2^* = 40.3% and heterogeneity Chi-square *p* = 0.070, random-effects OR: 0.49, 95% CI: 0.36–0.66, *p* < 0.001). A higher implantation depth was achieved in the COP group compared with the TCC group [standardized mean difference (SMD) = −0.324, 95% CI: (−0.469, −0.180)]. There was no significant difference between the two groups in second valve implantation, prosthesis pop-out, fluoroscopic time, post-operative left bundle branch block, mortality, stroke, moderate/severe paravalvular leakage, mean gradient, and length of hospital stay. However, radiation doses were higher in the COP group [SMD = 0.394, 95% CI: (0.216, 0.572), *p* < 0.001].

**Conclusion:**

In self-expanding TAVR, the application of the cusp overlap projection technique was associated with a lower risk of PPI compared with the standard TCC technique.

**Systematic review registration:**

[https://inplasy.com/inplasy-2022-4-0092/], identifier [INPLASY202240092].

## Introduction

As the PARTNER 3 study and Evolut Low-Risk study provided favorable evidence for transcatheter aortic valve replacement (TAVR) in low surgical risk patients (1, 2), updated guidelines on valvular heart disease included the recommendation of TAVR even for low-risk patients (3, 4). An increasing number of young patients with low surgical risk could be suitable candidates for TAVR. Permanent pacemaker implantation (PPI) is a common complication after TAVR. On the one hand, PPI after TAVR was thought to hamper the recovery of heart function. Also, recent studies highlighted that PPI was associated with worse clinical outcomes, including mortality and heart failure re-hospitalization (5). On the other hand, a pacemaker would need to be replaced due to battery depletion, which would obviously influence patients’ lifetime management, especially in relatively younger patients. Therefore, exploring new ways to reduce the incidence of PPI is important for the future development of TAVR.

The reported incidences of PPI before discharge are 5.9–32.0%, which show great variability among different centers (5). PPI has many predictors, including pre-existing right bundle branch block, atrioventricular block, short membranous septum length, left ventricular outflow calcification, deep prosthesis implantation depth, larger oversizing ratio, and similar (5, 6). However, most factors are not modifiable except for implantation depth and the oversizing ratio of the prosthesis (7). Since reducing the oversizing ratio might cause more paravalvular leakage (8, 9), a higher prosthesis implantation seems to be a good choice for reducing the risk of PPI.

**FIGURE 1:**
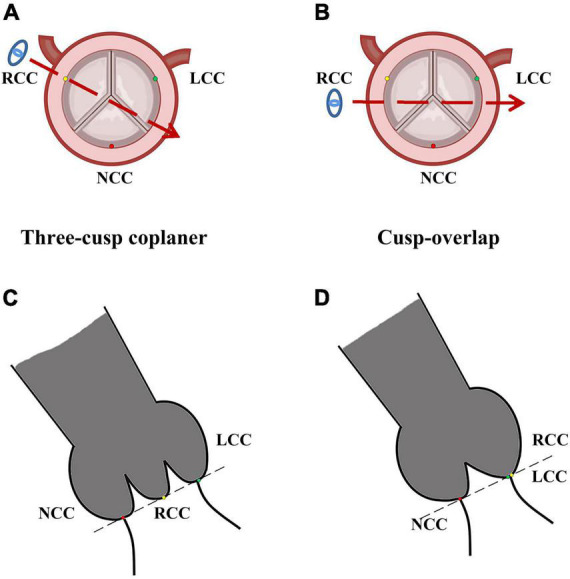
Image of cusp-overlap projection and three-cusp coplanar view. LCC, left coronary cusp; NCC, non-coronary cusp; RCC, right-coronary cusp. Typical image of standard three-cusp coplanar in pre-operative axial reconstruction **(A)** and fluoroscopy during TAVR procedure **(C)**. In standard fluoroscopy, RCC (yellow dot) was located in the middle of NCC (red dot), and LCC (green dot). Panels **(B,D)** represented images of cusp-overlap projection, where RCC (yellow dot) and LCC (green dot) were overlapped in fluoroscopy. The blue eye and dotted red arrows represented projection direction in fluoroscopy.

The current manufacturer recommends the implantation depth for self-expanding Evolut series prosthesis to be 3–5 mm. Higher prosthesis implantation means the distance between the annulus and lowest plane of metal stent needs to be less than 5 mm, or even 3 and 2 mm, which might increase the risk of the prosthesis pop-out. Actually, it is hard to precisely release the prosthesis higher using the conventional device implantation technique. Therefore, exploring new ways to achieve a higher prosthesis release is of essential importance. Cusp-overlap projection (COP), which is a new projection technique during prosthesis release, might be a viable solution. Unlike the standard three-cusp coplanar (TCC) view in which the right coronary cusp (RCC) is in the middle of the left coronary cusp (LCC) and non-coronary cusp (NCC), COP makes the LCC and the RCC overlap in order to eliminate parallax (Figure 1). It helps elongate the left ventricular outflow tract and evaluate the true releasing depth in NCC, thus precisely achieving higher implantation (10). However, as a new technique, it is uncertain whether COP is actually associated with higher implantation depth, less LBBB, and less PPI after the procedure. The aim of the current study was to perform a systematic review and meta-analysis to compare the COP and TCC.

## Methods

### Search strategy

A systematic literature search of PubMed and EMBASE database was conducted from the inception of these two databases (EMBASE from 1974 and PubMed from 1966) to 16 April 2022, following the Preferred Reporting Items for Systematic Reviews and Meta-Analyses (PRISMA) statement. The following terms were used for searching: (*TAVR* OR *TAVI* OR “*transcatheter aortic valve*”) AND (“*cusp overlap*” OR “*cusp overlapping*”). The title and abstract were screened by two independent investigators (YC and GZ), and the study eligibility and the quality were assessed. The disagreements were solved by consulting a third investigator (LW), following which a consensus was reached between three investigators. The study protocol was registered in INPLASY (INPLASY202240092).

### Study selection

The research meets the following criteria was included: (1) the study made a comparison between the cusp-overlap technique and the standard technique; (2) self-expanding valves were used; (3) at least 10 patients were enrolled in the study; (4) published in the English language; and (5) conference presentation and abstract fulfilling the above criteria. Case reports or single-arm studies were excluded. If the same population was reported in different studies, only the largest and the most recent studies were included.

### Outcomes of interest and data extraction

As our aim was to evaluate the efficacy and safety of the COP technique, the primary outcome of interest was post-operative (including in-hospital and 30-day) PPI. Secondary outcomes of interest were second valve implantation, prosthesis pop-out, intra-procedural radiation doses, fluoroscopic time, prosthesis implantation depth, length of hospital stay, and post-operative (including in-hospital and 30-day) LBBB, mortality, stroke, mean aortic valve gradient, and moderate or severe paravalvular leakage. Baseline and procedural characteristics were also gathered based on pre-defined extraction lists, including age, sex, surgical risk (Society of Thoracic Surgeon score or European System for Cardiac Operative Risk Evaluation score), pre-existing atrial fibrillation, LBBB, right bundle branch block, access of procedure, pre-dilatation, and post-dilatation. YC and GZ independently assessed the accuracy of the data and reached a consensus when discrepancies occurred.

### Statistical analysis

The results of the meta-analysis were summarized as odds ratio (OR), risk ratio, and 95% confidence interval (CI). Data presented as median (interquartile range) in the included studies were converted to mean ± SD, as described in previous studies (11, 12). The Cochrane *Q*-statistic (*χ^2^*) and Higgins’ and Thompson’s *I*^2^ statistics were assessed to test heterogeneity. A random-effect model was used if there was significant heterogeneity, which was defined as *I*^2^ > 50% or *p* ≤ 0.01. Otherwise, a fixed-effect model was used. A subgroup analysis was performed for PPI: data based on original article or conference presentation. Besides, a sensitivity analysis was also performed for PPI by removing each study from the pooled analysis. A two-sided *p* < 0.05 was considered to be statistically significant. The statistical analyses were performed with stata software version 14.0 (StataCorp LP, College Station, United States) using *metan* command.

### Quality and risk of bias assessment

Since all studies included in this meta-analysis were observational cohort studies, the quality of studies was assessed using Newcastle-Ottawa Scale. Publication bias for PPI was evaluated by the Begg test/Egger test. A funnel plot was also presented to visually evaluate publication bias.

## Results

### Included studies and study details

**FIGURE 2:**
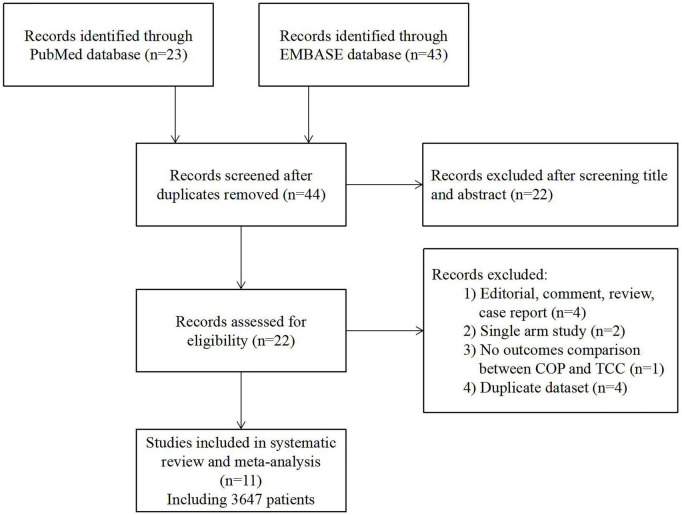
Flow diagram. COP, cusp-overlap projection; TCC, three-cusp coplanar.

The flowchart of the literature search is presented in Figure 2. A total of 11 studies, including 3,647 patients, were finally included in this meta-analysis (13–23). All of the included studies were non-randomized cohort studies. Three studies were original articles, while the others were conference presentations or abstracts. The Newcastle-Ottawa Scale score ranged from 6 to 9 points, revealing a relatively low risk of bias (Supplementary Table 1). Across the 11 selected reports, 1,453 and 2,194 patients underwent self-expanding TAVR using COP or TCC technique, respectively. Detail of the studies’ baseline characteristics (including age, sex, surgical risk, pre-existing arrhythmia, etc.) and procedural characteristics are shown in Table 1 and Supplementary Table 2.

**TABLE 1:** Overview of the included study.

References	Study type	Region	Year	Patients, *n*	Age, years	Female	Surgical risk
Doldi et al. (21)	Original article	Germany (Munich)	COP: December 2020 to October 2021 TCC: April 2019 to November 2020	COP: 61 TCC: 61	COP: 82.5 (79.0, 86.8) TCC: 83.3 (80.4, 87.9)	COP: 15/61 TCC: 10/61	STS: COP: 3.5 (2.6, 3.7) TCC: 3.1 (2.0, 4.9)
Pascual et al. (22)	Original article	Spain Canada Mexico	February 2015 to February 2021	COP: 161 TCC: 161	COP: 81.8 ± 8.9 TCC: 82.5 ± 6.9	COP: 82/161 TCC: 91/161	STS: COP: 4.3 ± 1.9 TCC: 4.2 ± 2.0
Medranda et al. (13)	Abstract	United States (Washington and Baltimore)	January 2018 to June 2021	COP: 52 TCC: 225	Mean age: 81.5	160/277	Mean STS score: 4.5
Mendiz et al. (23)	Original article	Argentina Slovenia Costa Rica	COP: August 2019 to June 2020 TCC: before August 2019	COP: 156 TCC: 101	COP: 79.6 ± 7.4 TCC: 79.8 ± 7.9	COP: 77/156 TCC: 52/101	STS: COP: 5.9 ± 2.6 TCC: 5.8 ± 2.4
Sadiq et al. (14)	Abstract	United States (Connecticut)	COP: January 2020 to January 2021 TCC: January 2019 to January 2020	COP: 75 TCC: 104	–	–	–
Perez et al. (15)	Abstract	Chile	–	COP: 41 TCC: 32	COP: 78.3 ± 6.9 TCC: 77.7 ± 10.0	COP: 26/41 TCC: 21/32	STS: COP: 4.3 ± 2.5 TCC: 6.7 ± 3.9
Maier et al. (16)	Abstract	Germany (Dusseldorf and Frankfurt)	COP: September 2020 to February 2021 TCC: January 2016 to August 2020	COP: 127 TCC: 589	–	–	–
Goel et al. (17)	Abstract	United States (New York)	January 2017 to January 2021	COP: 202 TCC: 325	–	–	–
Jones et al. (18)	Abstract	Australia	COP: January 2020 to March 2021 TCC: between 2008 and 2021	COP: 139 TCC: 452	81 ± 13	–	EuroSCORE II 5.2 ± 4.1
Aljabbary et al. (19)	Abstract	Canada	COP: July 2018 to February 2020 TCC: April 2016 to March 2017	COP: 393 TCC: 127	–	–	–
Raza et al. (20)	Abstract	United States (New York)	November 2014 to June 2016	COP: 46 TCC: 17	COP: 81 ± 9 TCC: 77 ± 9	COP: 23/46 TCC: 6/17	STS: COP: 9.1 ± 5.8 TCC: 7.7 ± 4.6

COP, cusp-overlap projection; EuroSCORE, European System for Cardiac Operative Risk Evaluation; STS, Society of Thoracic Surgeons; TCC, three-cusp coplanar.

### Primary outcomes

**FIGURE 3:**
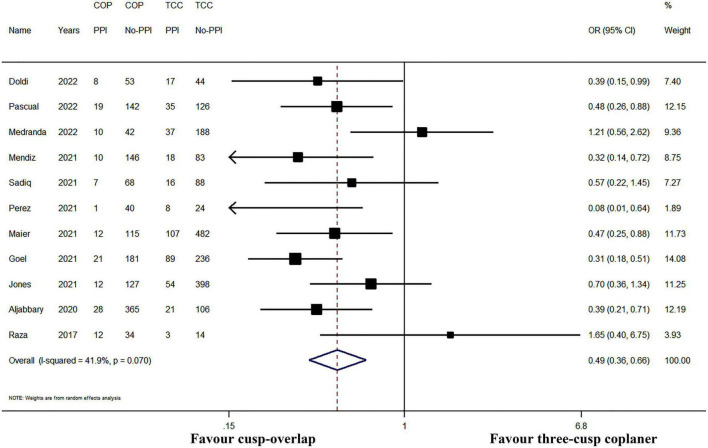
Meta-analysis of the primary outcome of interest. CI, confidence interval; COP, cusp-overlap projection; OR, odds ratio; PPI, permanent pacemaker implantation; TCC, three-cusp coplanar. Forrest plot has shown the comparison of post-operative PPI. Since significant heterogeneity existed (heterogeneity Chi-square *p* = 0.070), a random-effects model was used. The bars represented a 95% CI.

All the included studies have reported the incidence of PPI after TAVR. The incidence of post-operative PPI ranged from 2.4 to 26.1% in the COP group, while the PPI rate ranged between 11.9 and 27.9% in the TCC group. In pooled analysis, the cumulative PPI incidence was 9.3% (95% CI: 6.9–11.7%, random-effects model, *I^2^* = 57.6%, heterogeneity Chi-square *p* = 0.009) and 18.9% (95% CI: 15.5–22.3%, random-effects model, *I^2^* = 72.2%, heterogeneity Chi-square *p* < 0.001) in COP group and TCC group, respectively. The application of the COP technique was associated with a significant PPI risk reduction (*I^2^* = 40.3% and heterogeneity Chi-square *p* = 0.070, random-effects OR: 0.49, 95% CI: 0.36–0.66, *p* < 0.001; Figure 3).

### Secondary outcomes

**FIGURE 4:**
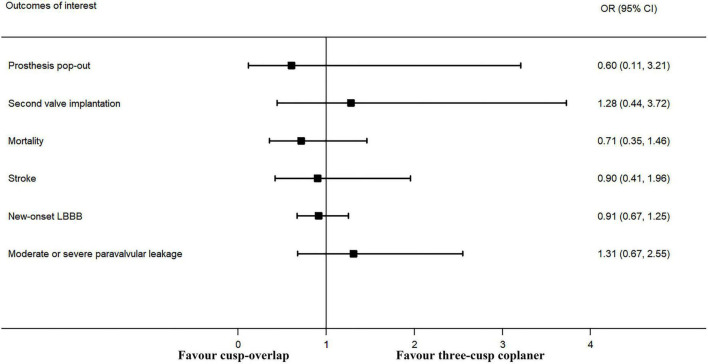
Meta-analysis of secondary outcomes of interest (categorical variables). LBBB, left bundle branch block. Forrest plot has shown the comparison of secondary outcomes of interest, including prosthesis pop-out, second valve implantation, post-operative mortality, post-operative stroke, post-operative new-onset LBBB, and moderate or more paravalvular leakage after TAVR procedure. The heterogeneity was not significant in any of the analyses, and a fixed-effects model was used. The bars represented a 95% CI.

**FIGURE 5:**
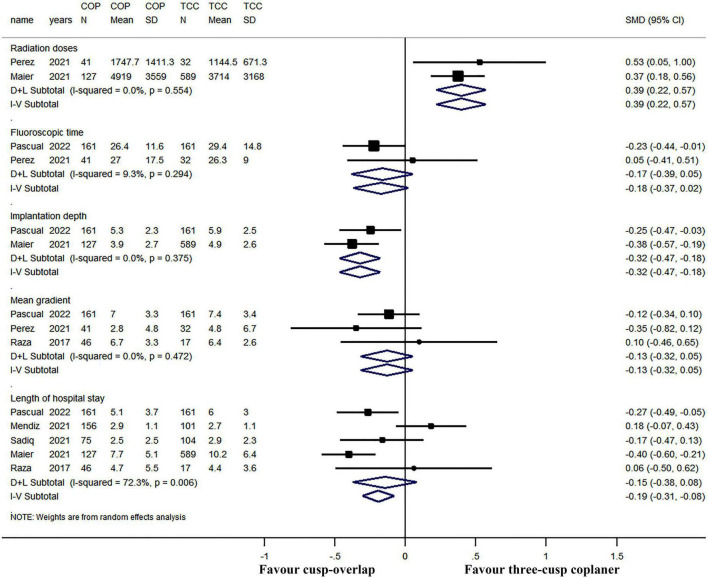
Meta-analysis of secondary outcomes of interest (continuous variables). Both the random-effects model (D + L) and fixed-effects model (I–V) have been presented in the Forrest plot. Based on heterogeneity examination, radiation doses, fluoroscopic time, implantation depth, and mean gradient were tested using the fixed-effects model, while the length of hospital stay should be compared using a random-effects model. The pooled analyses suggested that a higher implantation depth was achieved by using the COP technique, while radiation doses were higher in the COP group. The bars represented a 95% CI.

The forest plot and details of the analyses of the secondary outcomes are shown in Figures 4,5 and Tables 2,3. Using COP technique, a higher implantation depth was achieved [standardized mean difference (SMD) = −0.324, 95% CI: (−0.469, −0.180)]. There were no significant differences between the two groups in second valve implantation, prosthesis pop-out, fluoroscopic time, post-operative LBBB, mortality, stroke, moderate/severe paravalvular leakage, mean aortic valve gradient, and length of hospital stay. However, radiation doses were higher in COP group [SMD = 0.394, 95% CI: (0.216, 0.572), *p* < 0.001].

**TABLE 2:** Secondary outcomes of the meta-analysis (continuous variables).

	COP group		TCC group	
**Radiation doses**	***n***	**Mean ± SD**	***n***	**Mean ± SD**
Perez et al. (15)	41	1,747.7 ± 1,411.3	32	1,144.5 ± 671.3
Maier et al. (16)	127	4,919 ± 3,559	589	3,714 ± 3,168
Heterogeneity: χ^2^ = 0.35, df = 1 (*p* = 0.554), *I*^2^ = 0%. Fixed-effects model, SMD: 0.394, 95% CI: (0.216, 0.572), *p* < 0.001.				
**Fluoroscopic time**	***n***	**Mean ± SD**	***n***	**Mean ± SD**
Pascual et al. (22)	161	26.4 ± 11.6	161	29.4 ± 14.8
Perez et al. (15)	41	27.0 ± 17.5	32	26.3 ± 9
Heterogeneity: χ^2^ = 1.10, df = 1 (*p* = 0.294), *I*^2^ = 9.3%. Fixed-effects model, SMD: −0.175, 95% CI: (−0.373, 0.023), *p* = 0.083.				
**Implantation depth**	***n***	**Mean ± SD**	***n***	**Mean ± SD**
Pascual et al. (22)	161	5.3 ± 2.3	161	5.9 ± 2.5
Maier et al. (16)	127	3.9 ± 2.7	589	4.9 ± 2.6
Heterogeneity: χ^2^ = 0.79, df = 1 (*p* = 0.375), *I*^2^ = 0%. Fixed-effects model, SMD: −0.324, 95% CI: (−0.469, −0.180), *p* < 0.001.				
**Mean gradient**	***n***	**Mean ± SD**	***n***	**Mean ± SD**
Pascual et al. (22)	161	7.0 ± 3.3	161	7.4 ± 3.4
Perez et al. (15)	41	2.8 ± 4.8	32	4.8 ± 6.7
Raza et al. (20)	46	6.7 ± 3.3	17	6.4 ± 2.6
Heterogeneity: χ^2^ = 1.50, df = 2 (*p* = 0.472), *I*^2^ = 0%. Fixed-effects model, SMD: −0.132, 95% CI: (−0.319, 0.054), *p* = 0.165.				
**Length of hospital stay**	***n***	**Mean ± SD**	***n***	**Mean ± SD**
Pascual et al. (22)	161	5.1 ± 3.7	161	6.0 ± 3.0
Mendiz et al. (23)	156	2.9 ± 1.1	101	2.7 ± 1.1
Sadiq et al. (14)	75	2.5 ± 2.5	104	2.9 ± 2.3
Maier et al. (16)	127	7.7 ± 5.1	589	10.2 ± 6.4
Raza et al. (20)	46	4.7 ± 5.5	17	4.4 ± 3.6
Heterogeneity: χ^2^ = 14.42, df = 4 (*p* = 0.006), *I*^2^ = 72.3%. Random-effects model, SMD: −0.148, 95% CI: (−0.377, 0.082), *p* = 0.207.				

COP, cusp-overlap projection; SD, standard difference; SMD, standard mean difference; TCC, three-cusp coplanar. A random-effects model was used if significant heterogeneity existed. Otherwise the fixed-effects model was used.

**TABLE 3:** Secondary outcomes of the meta-analysis (categorical variables).

	COP group events/patients	TCC group events/patients
**Prosthesis pop-out**		
Pascual et al. (22)	1/161	3/161
Mendiz et al. (23)	1/156	0/101
Raza et al. (20)	0/46	0/17
Heterogeneity: χ^2^ = 1.36, df = 1 (*p* = 0.244), *I*^2^ = 26.3%. Fixed-effects model, OR: 0.602, 95% CI: 0.113–3.206, *p* = 0.584.		
**Second valve implantation**		
Doldi et al. (21)	1/61	0/61
Pascual et al. (22)	5/161	5/161
Raza et al. (20)	2/46	0/17
Heterogeneity: χ^2^ = 0.50, df = 2 (*p* = 0.779), *I*^2^ = 0%. Fixed-effects model, OR: 1.276, 95% CI: 0.437–3.722, *p* = 0.655.		
**Mortality**		
Doldi et al. (21)	0/61	1/61
Pascual et al. (22)	4/161	4/161
Mendiz et al. (23)	4/156	5/101
Goel et al. (17)	3/202	8/325
Raza et al. (20)	3/46	0/17
Heterogeneity: χ^2^ = 1.57, df = 4 (*p* = 0.815), *I*^2^ = 0%. Fixed-effects model, OR: 0.714, 95% CI: 0.350–1.458, *p* = 0.356.		
**Stroke**		
Doldi et al. (21)	2/61	3/61
Pascual et al. (22)	9/161	9/161
Mendiz et al. (23)	1/156	0/101
Raza et al. (20)	1/46	1/17
Heterogeneity: χ^2^ = 0.80, df = 3 (*p* = 0.849), *I*^2^ = 0%. Fixed-effects model, OR: 0.900, 95% CI: 0.414–1.955, *p* = 0.790.		
**New-onset LBBB**		
Doldi et al. (21)	29/61	27/61
Pascual et al. (22)	33/161	27/161
Mendiz et al. (23)	9/156	13/101
Goel et al. (17)	24/202	47/325
Heterogeneity: χ^2^ = 5.16, df = 3 (*p* = 0.160), *I*^2^ = 41.9%. Fixed-effects model, OR: 0.913, 95% CI: 0.668–1.249, *p* = 0.569.		
**Moderate or more paravalvular leakage**		
Doldi et al. (21)	3/61	1/61
Pascual et al. (22)	6/161	7/161
Mendiz et al. (23)	4/156	2/101
sadiq et al. (14)	7/75	5/104
Raza et al. (20)	1/46	1/17
Heterogeneity: χ^2^ = 2.47, df = 4 (*p* = 0.651), *I*^2^ = 0%. Fixed-effects model, OR: 1.310, 95% CI: 0.673–2.549, *p* = 0.426.		

The abbreviation and application of the random- or fixed-effects model were the same as in Table 2. The pooled analysis of post-operative outcomes included studies that reported in-hospital or 30-day outcomes.

### Publication bias

**FIGURE 6:**
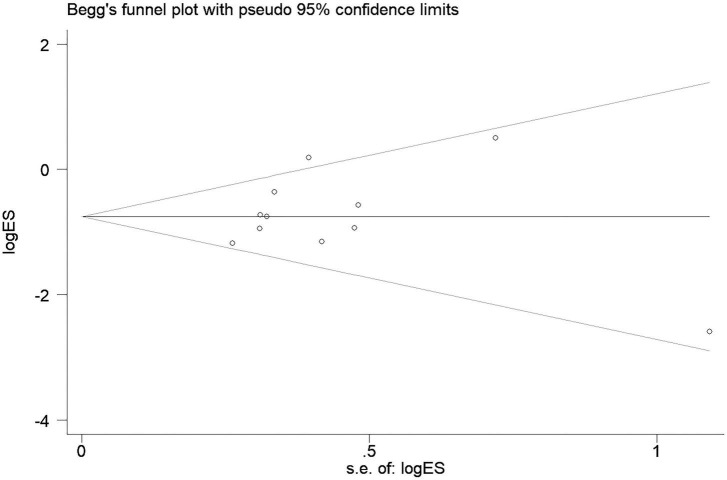
Begg’s funnel plot for the publication bias. Shape of the funnel plot showed no obvious asymmetry. Besides, both Egger’s test (*p* = 0.764) and Begg’s test (*p* = 0.436) showed that no significant publication bias existed.

The funnel plot is presented in Figure 6, with no obvious asymmetry. Besides, both Egger’s test (*p* = 0.764) and Begg’s test (*p* = 0.436) showed that no significant publication bias existed.

### Sensitivity analysis

**FIGURE 7:**
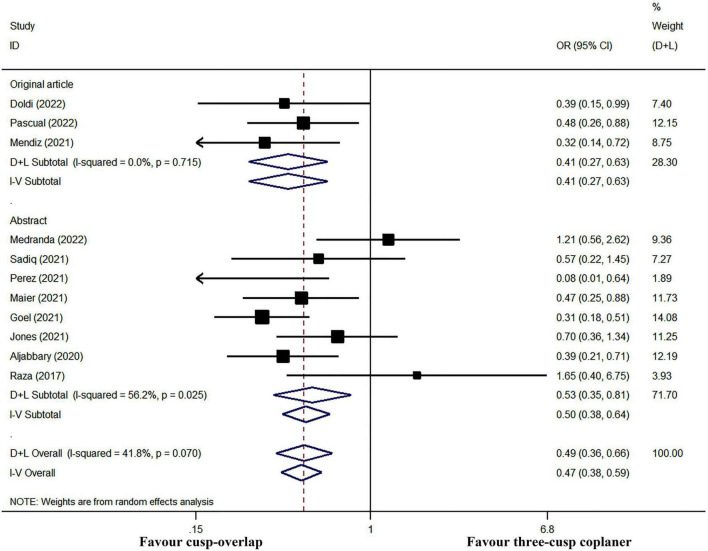
Subgroup meta-analysis by study type. Both the random-effects model (D + L) and fixed-effects model (I–V) have been presented in the Forrest plot. Significant heterogeneity existed in studies reported in the abstract, while there was no obvious heterogeneity in a subgroup analysis of original article studies. Therefore, the fixed-effects model should be performed in a meta-analysis of post-operative PPI in three original articles, and the random-effects model should be used in subgroup analysis in abstract studies. However, the COP group had a significantly lower risk of PPI in either subgroup meta-analysis.

**FIGURE 8:**
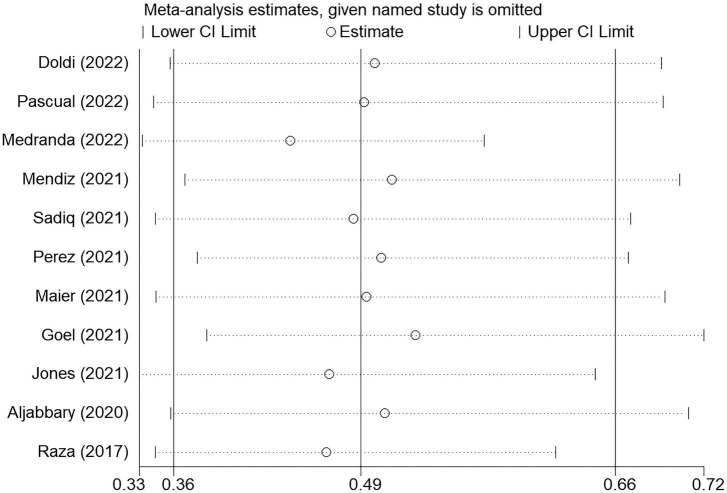
Sensitive analysis through sequentially omitting each study from the pooled analysis. The effect size remained stable when any of the studies were omitted. The application of the COP technique was associated with approximately half PPI risk reduction in all models.

In subgroup analyses, PPI risk was still significantly lower in COP group when only pooled data of original articles (fixed-effect OR: 0.41, 95% CI: 0.27–0.63, *p* < 0.001; *I^2^* = 0% and heterogeneity Chi-square *p* = 0.715) or conference abstracts (random-effect OR: 0.53, 95% CI: 0.35–0.81, *p* = 0.003; *I^2^* = 56.2% and heterogeneity Chi-square *p* = 0.025, Figure 7) were used. Furthermore, the sensitivity analysis was performed by sequentially omitting each study from the pooled analysis using a random-effects model (Figure 8). The effect size remained stable when any of the studies were omitted. The application of the COP technique was associated with a lower risk of PPI in all models.

## Discussion

To our knowledge, the present study is the first systematic review and meta-analysis that evaluated the safety and efficacy of the cusp-overlap technique. The main finding of the study are: (1) compared with standard TCC projection, the COP technique was associated with less PPI after self-expanding TAVR; (2) a higher implantation depth could be reached by using the COP technique; (3) the risk of post-operative LBBB, mortality, stroke, moderate or severe paravalvular leakage, and the mean gradient after TAVR were comparable between COP and TCC technique; and (4) however, the radiation doses were higher in the COP group.

### Cusp-overlap reduce permanent pacemaker implantation after self-expanding transcatheter aortic valve replacement

Although TAVR was thought to have similar or better mortality and stroke outcomes than surgical aortic valve replacement (SAVR), the risk of PPI after TAVR was more than twofold greater than SAVR (24–26). Self-expanding valve, which is widely used worldwide, was proven to be associated with a higher risk of PPI compared with a balloon-expandable valve. Even if in the last ten years great advances have been made in devices and techniques, there were still 17.4% of patients who underwent PPI within 30 days after self-expanding TAVR in the Evolut Low-Risk Trial (2), while the incidence of 30-day PPI was only 6.6% in patients who underwent balloon-expandable TAVR in PARTNER 3 study (1).

Since the conduction system such as the bundle of His and left bundle branch are located in the membranous septum just beneath the annulus plane, it is widely acknowledged that a higher prosthesis implantation depth could reduce PPI risk. Nevertheless, it is challenging for operators to precisely release prosthesis within a margin of millimeters.

Cusp-overlap projection technique, which was firstly reported at a conference in 2016, was introduced systematically in 2018 (27, 28). COP technique rapidly became the focus of research all over the world. Nine studies published between 2020 and 2022 have compared COP with standard TCC technique. Recently, a well-designed propensity score matching study also reported a significantly lower PPI risk after TAVR (relative risk: 0.54) (21). As a current topic of interest, COP is considered as a great procedural improvement and has even been recommended as the standard implantation technique for self-expanding TAVR (29). However, the evidence on COP is still relatively scarce. Therefore, we conducted this meta-analysis, finding that COP reduced over 45% risk of post-operative PPI (risk ratio: 0.544, 95% CI: 0.418–0.708, *p* < 0.001, random effects model, Supplementary Figure 1), proving the efficacy of COP technique in self-expanding TAVR.

Normally, a standard TCC view is suitable for the balloon-expandable valve. The balloon-expandable prosthesis is more easily located in the center of the aortic annulus and could often be released perpendicular to the annulus. However, the releasing process of the self-expanding valve was different. Since the self-expanding valve had an obvious longer stent frame, the prosthesis would first contact NCC. Accordingly, the NCC side served as a support point, and the prosthesis naturally progressed toward to the outer curvature of the aortic root (29). Finally the prosthesis contacted the LCC side, after which the valve releasing was finished. Therefore, the deployment of self-expanding was usually asymmetric, and the visibility of the NCC side hinge point was found to be important. In the COP technique, besides the three coronary cusps in the same plane, the LCC and RCC overlapped during fluoroscopy (Figure 1), which could facilitate better visualization of NCC and remove the parallax of a delivery system. Moreover, the conduction system and membranous septum, which are just located between the NCC and RCC, could also be better visualized. Besides, the left ventricular outflow tract was elongated, and the implantation depth could be evaluated more accurately. Considering the above, a lower PPI incidence was achieved using the COP technique.

### Cusp-overlap and additional peri-procedural risk

Since the aim of the COP technique was to achieve higher prosthesis implantation, the risk of prosthesis pop-out, paravalvular leakage, second valve implantation, and whether this technique would lead to additional peri-procedural risk were the main issues. In the present study, the risk of paravalvular leakage, second valve implantation, and clinical events such as mortality and stroke were similar between COP and standard TCC groups. However, although only 2 out of 363 patients who received the COP technique suffered prosthesis pop-out in the included studies, Maier et al. suggested that COP was associated with higher rates of device reposition compared to the standard technique (57.8 vs. 16.2%, *p* < 0.001) in their center (30). Besides, it should be noticed that the radiation doses were higher in the COP group. Even if this might be related to the learning curve of a new technique, the difficulty of achieving higher prosthesis implantation and the potential additional risk of COP needs to be further studied.

### Future of cusp-overlap projection technique

Higher implantation of a self-expanding prosthesis might lead to coronary occlusion. Prevention techniques such as chimney stent protection and BASILICA (Bioprosthetic or native Aortic Scallop Intentional Laceration to prevent Iatrogenic Coronary Artery obstruction during TAVR) can be considered for patients at high risk of coronary occlusion. Besides, coronary access availability is another concern when using the COP technique to implant prosthesis higher (31). The method used to achieve commissure alignment during TAVR is another new technique, intended to make coronary ostia more easily available (32–34). In the SAVR procedure, surgeons can directly see coronary ostia and align the bioprosthesis with native valve commissures. However, this is quite challenging during the TAVR procedure. Recently, the COP technique was thought to be a feasible solution to achieve commissure alignment (34, 35). Using COP technique, the left side of fluoroscopy was aligned to the midpoint of NCC, and the commissure could be better visualized. However, the relevant studies are scarce and further evaluation of commissure alignment using COP versus TCC is needed.

Ultimately, COP could significantly reduce the risk of PPI. The incidence of PPI after self-expanding TAVR was only 6.4% in a low-volume center where the COP technique was used (23). The preliminary data from Optimize PRO study (NCT04091048), which evaluated the COP technique in Evolut PRO and PRO+, also revealed a favorable PPI rate (8.8%) (10). Besides, the incidence of PPI could be reduced by membranous septum guided prosthesis implantation, which could also be suitable when using the COP technique (6, 36). Theoretically, the combination of these two techniques might further reduce PPI risk. It was worth anticipating that the risk of PPI would be obviously reduced with the application of the COP technique, and the incidence of PPI after self-expanding TAVR could be as low as balloon-expandable valve implantation and even comparable to SAVR in the future.

## Limitations

The main limitation of this study is the non-randomized design of the included studies, which means baseline characteristics could not be matched. Secondly, only three original articles were included, while the remaining studies were conference presentations and abstracts. However, similar results can be found in the subgroup analyses by study type. There was no significant publication bias in our study, as conference abstracts were also included. Given the above, based on the obtained results, we can still deduce that COP can reduce PPI risk in self-expanding TAVR. Besides, so many studies of COP have been carried out within five years, revealing that the COP technique is currently a topic of great interest. A large number of conference reports make this systematic review more warranted and the importance of our study is highlighted. Third, the standard TCC technique was performed earlier than the COP technique in half of the included studies, which could lead to obvious selection bias. Since TAVR is a rapidly changing technique, recently treated patients were more likely to benefit from new devices and technological advances. Nevertheless, the current PPI incidence in patients using COP technique may also have been influenced by the learning curve effect. Forth, different centers could use the COP technique with different operative tips (such as starting level, rapid pacing, fluoroscopic strategy, etc.). As we could not fully obtain this information, this might lead to bias. Finally, some endpoints such as acute kidney injury and vascular complications have not been adequately reported in the included studies. Future large-scale, multi-center, and randomized controlled studies are needed to further evaluate the safety of the COP technique.

## Conclusion

In self-expanding TAVR, the COP technique was associated with a significant risk reduction of post-operative PPI. COP technique could be more widely applied in clinical practice, with the caution of higher radiation doses. However, since COP is a new technique, more large-scale and high-quality evidence is needed.

## Data availability statement

The original contributions presented in this study are included in the article/Supplementary material, further inquiries can be directed to the corresponding authors.

## Author contributions

YC, GZ, and XL proposed the idea for the study and finished the study design. YC and GZ retrieved the studies, collected and extracted the data with disagreements resolved by LW. YC, WW, HC, and MT performed the statistical analyses and drafted the manuscript. DK and YL reviewed and revised the manuscript. All authors contributed to the preparation, read, and approved the final manuscript.
