# Supplementary material for: Comparison of cusp-overlap projection and standard three-cusp coplanar view during self-expanding transcatheter aortic valve replacement: A systematic review and meta-analysis
Source: Front Cardiovasc Med. 2022 Aug 17;9:927642. doi: 10.3389/fcvm.2022.927642 (PMC9428452; doi:10.3389/fcvm.2022.927642)
Supplement: Supplementary file 1 [file Data_Sheet_1.docx]

**Supplementary Table 1. The risk of bias assessed by the Newcastle-Ottawa scale criteria.**

|  | Doldi | Pascual | Medranda | Mendiz | Sadiq | Perez | Maier | Goel | Jones | Aljabbary | Raza |
| --- | --- | --- | --- | --- | --- | --- | --- | --- | --- | --- | --- |
| **Selection** |  |  |  |  |  |  |  |  |  |  |  |
| -Representativeness (1) | 1 | 1 | 1 | 1 | 1 | 0 | 1 | 1 | 1 | 1 | 1 |
| -Non-exposed cohort (1) | 1 | 1 | 1 | 1 | 1 | 0 | 1 | 1 | 1 | 1 | 1 |
| -Exposure (1) | 1 | 1 | 1 | 1 | 1 | 1 | 1 | 1 | 1 | 1 | 1 |
| -Outcomes (1) | 1 | 1 | 0 | 1 | 0 | 1 | 0 | 1 | 0 | 0 | 0 |
| **Comparability** |  |  |  |  |  |  |  |  |  |  |  |
| -Most important factor (1) | 1 | 1 | 0 | 1 | 0 | 1 | 0 | 0 | 0 | 0 | 0 |
| -Additional factor (1) | 1 | 1 | 0 | 1 | 0 | 1 | 0 | 0 | 0 | 0 | 0 |
| **Outcome** |  |  |  |  |  |  |  |  |  |  |  |
| -Assessment (1) | 1 | 1 | 1 | 1 | 1 | 1 | 1 | 1 | 1 | 1 | 1 |
| -Follow-up (1) | 1 | 1 | 1 | 1 | 1 | 1 | 1 | 1 | 1 | 1 | 1 |
| -% Follow-up (1) | 1 | 1 | 1 | 1 | 1 | 1 | 1 | 1 | 1 | 1 | 1 |
| Overall | 9 | 9 | 6 | 9 | 6 | 7 | 6 | 7 | 6 | 6 | 6 |

**Supplementary Table 2. Pre-existing arrhythmia and key procedural characteristics of the included studies**

| **First author, publication year** | **Atrial fibrillation** | **Prior RBBB** | **Prior LBBB** | **Transfemoral access** | **pre-dilatation** | **post-dilatation** |
| --- | --- | --- | --- | --- | --- | --- |
| Doldi et al, 2022 | COP: 26/61  TCC: 25/61 | COP: 11/61  TCC: 4/61 | COP: 9/61  TCC: 8/61 | COP: 61/61  TCC: 61/61 | - | - |
| Pascual et al, 2022 | COP: 37/161  TCC: 33/161 | COP: 22/161  TCC: 25/161 | COP: 22/161  TCC: 22/161 | COP: 161/161  TCC: 161/161 | COP: 24/161  TCC: 31/161 | COP: 43/161  TCC: 53/161 |
| Medranda et al, 2022 | - | - | - | - | - | - |
| Mendiz et al, 2021 | COP: 26/156  TCC: 16/101 | COP: 18/156  TCC: 10/101 | COP: 15/156  TCC: 10/101 | COP: 156/156  TCC: 101/101 | COP: 86/156  TCC: 58/101 | COP: 34/156  TCC: 25/101 |
| Sadiq et al, 2021 | - | - | - | - | - | - |
| Perez et al, 2021 | - | COP: 4/41  TCC: 1/32 | COP: 4/41  TCC: 2/32 | 72/73 | COP: 21/41  TCC: 7/32 | - |
| Maier et al, 2021 | - | - | - | - | - | - |
| Goel et al, 2021 | - | - | - | COP: 202/202  TCC: 325/325 | - | - |
| Jones et al, 2021 | - | - | - | - | - | - |
| Aljabbary et al, 2020 | - | - | - | - | - | - |
| Raza et al, 2017 | COP: 16/46  TCC: 6/17 | - | - | COP: 46/46  TCC: 15/17 | - | COP: 5/46  TCC: 0/17 |

COP: cusp-overlap projection; TCC: three-cusp coplaner

**Supplementary Figure 1. Meta-analysis of primary outcome of interest by risk ratio**

**
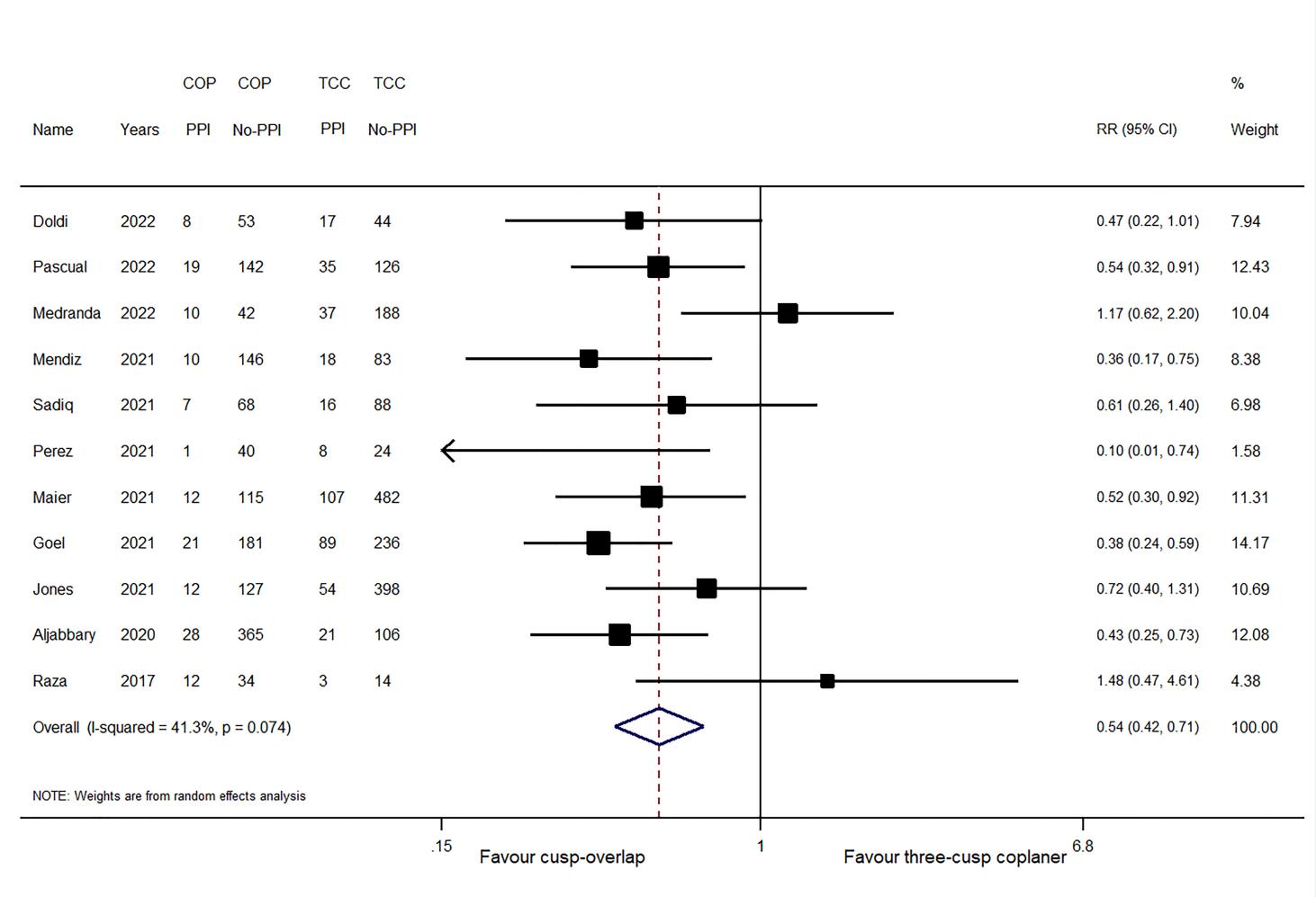
**

CI, confidence interval; COP, cusp-overlap projection; RR, risk ratio; PPI, permanent pacemaker implantation; TCC, three-cusp coplaner.

Since significant heterogeneity existed (heterogeneity chi-square p=0.074), random-effects model was used. The application of COP technique was associated with 45.6% risk reduction of post-operative PPI. The bars represented 95% confidence interval.
